# Metallic small y stent placement at primary right carina for bronchial disease

**DOI:** 10.1186/s12890-018-0742-1

**Published:** 2018-11-29

**Authors:** Yonghua Bi, Jindong Li, Zepeng Yu, Jianzhuang Ren, Gang Wu, Xinwei Han

**Affiliations:** 1 Department of Interventional Radiology, the First Affiliated Hospital of Zhengzhou University, Zhengzhou, China; 2 Department of Thoracic Surgery, the First Affiliated Hospital of Zhengzhou University, Zhengzhou, China

**Keywords:** Bronchial fistula, Bronchial stenosis, Sten, Primary right carina, Fluoroscopy

## Abstract

**Background:**

Metallic large Y stent placement has been used mainly for airway disease around the main carina. However, few studies have reported this treatment for bronchial disease around the primary right carina.

**Methods:**

Twenty-eight patients were treated by small y stent. All stents were custom-designed and placed under fluoroscopic guidance. Clinical and imaging data were analyzed retrospectively.

**Results:**

Thirty-one stents were successfully inserted in 28 patients. Twenty-five patients succeed at the first attempt (89.3%), and 3 patients needed a second attempt. Twelve complications occurred in 10 patients (35.7%). Stent restenosis and sputum retention were the most common complications. Five patients underwent successful stent removal due to complications or cure efficacy. During follow up, 17 patients died of tumors and one died of myocardial infarction. The 1-, 3-, and 5-year survival rates were 49.3, 19.6 and 19.6%, respectively.

**Conclusions:**

Metallic small y stent placement is technically feasible, effective and safe for bronchial disease around the primary right carina.

## Background

Metallic stent placement has been known as an effective treatment for patients with airway disease [1–10]. Metallic large Y stent placement has been used mainly for airway disease around the main carina [11, 12]. Clinically, bronchial disease is also common shown around the primary right carina. For example, silicone small y stents have been used to treat airway disease around the left carina [13]. Placement of metallic small y stents can be less traumatizing with the help of guide wires [14], and may well be an alternative to the silicone y stent [14]. Unfortunately, few studies have reported the use of metallic small y stents for the treatment of bronchial disease around primary right carina. In this study, we used the metallic y stent to treat bronchial disease that involved primary right carina, and aimed to determine the safety, feasibility and efficacy of this stenting technique.

## Materials and methods

### Patients

**Fig. 1:**
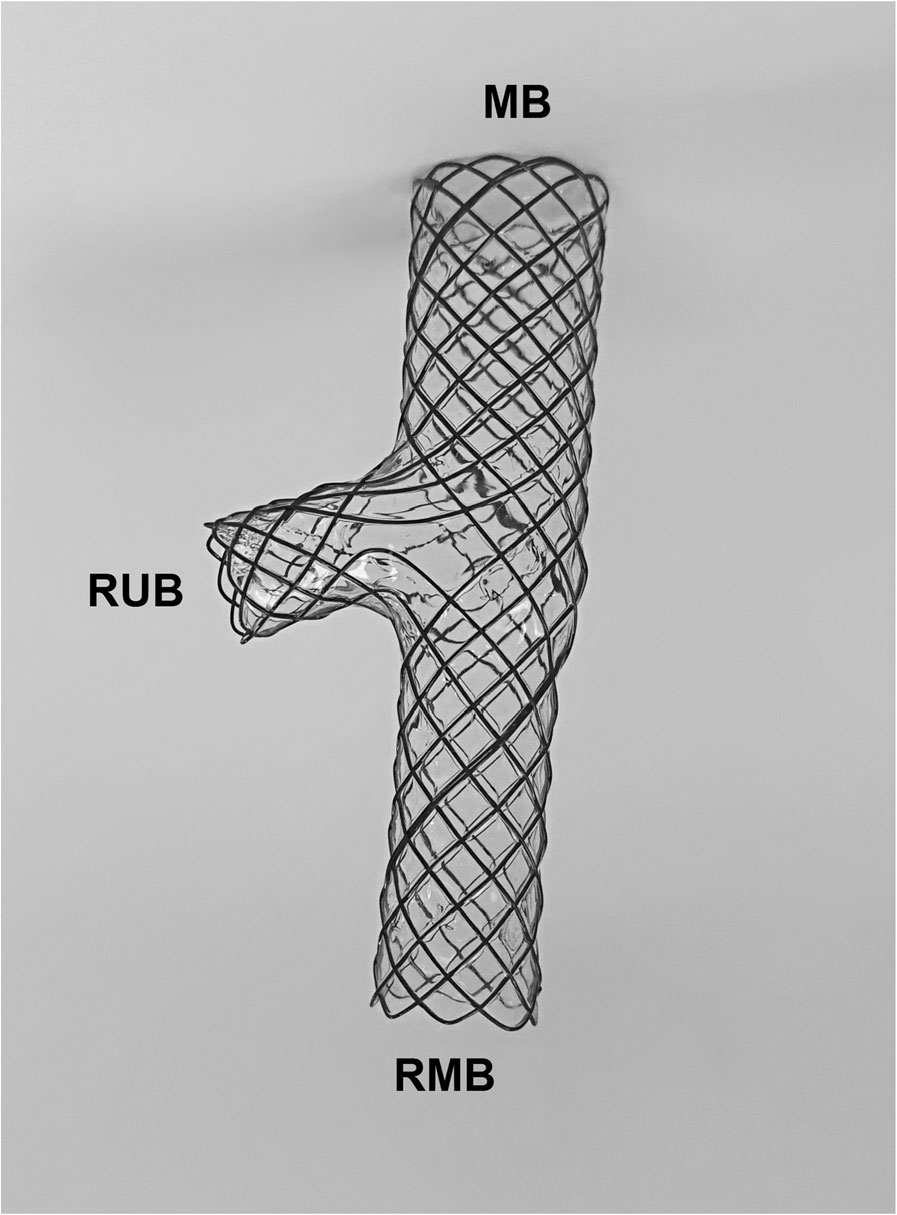
Photo of metallic airway stent. MB: main body; RUB: right up bronchus; RMB: right middle bronchus

**Fig. 2:**
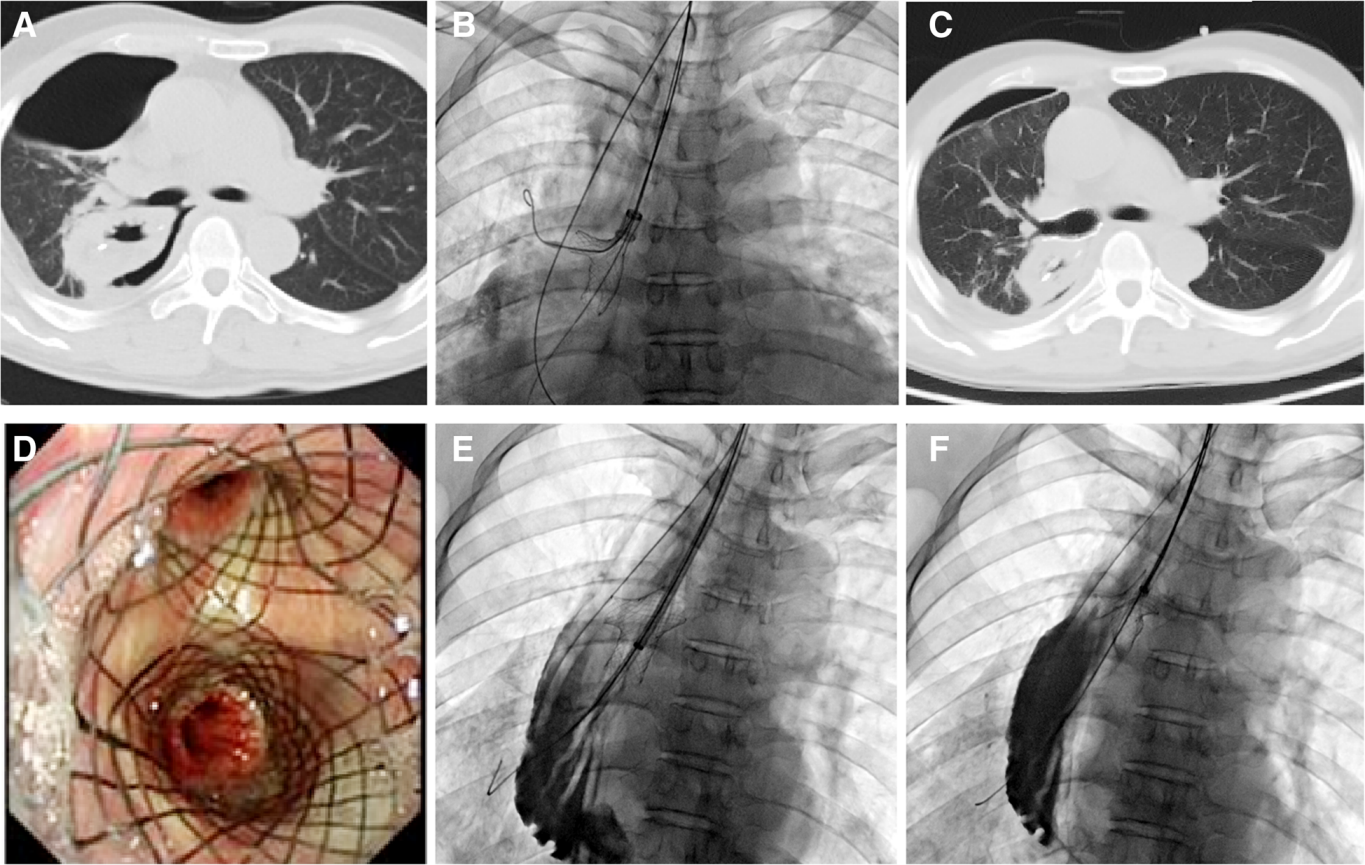
Treatment of a 59-year-old man with fistula in right main bronchus. a, Chest SCT showed fistula in right main bronchus with right pneumothorax. b, Small y covered stent inserted in primary right carina. c, Chest SCT shows sealing of bronchial fistula with decreased right pneumothorax. d, Bronchoscopy confirmed complete seal of the fistula. E, A long sheath was introduced for stent removal after 167 days due to patient cure. F, Tip of hook was placed next to end of stent and stent was withdrawn from the airway wall

**Fig. 3:**
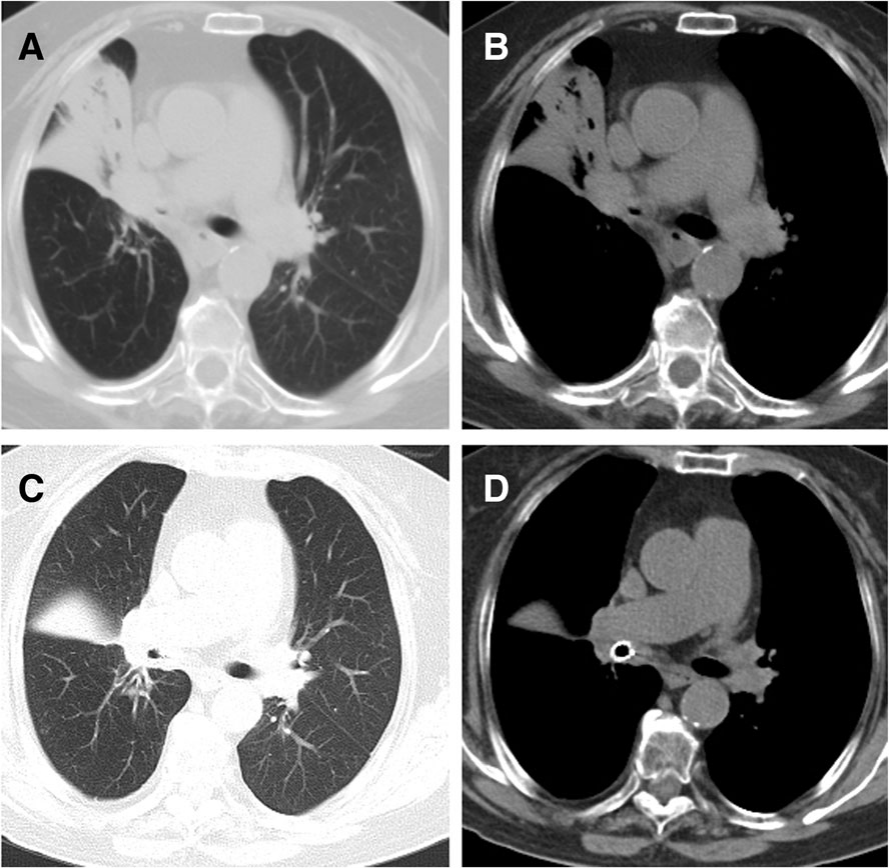
Treatment of a 77-year-old woman with severe stenosis in right main bronchus. a, b, Chest SCT showed severe stenosis in right main bronchus and atelectasis. c, d After small y stent placement, chest SCT confirmed patency of right main bronchus and disappearance of atelectasis

From January 2011 to May 2017, 28 patients were treated for bronchial disease by metallic small y stent in our department. Bronchoscopy and chest spiral computed tomography (SCT) was used for the diagnosis of bronchial disease. The medical records of these patients were retrospectively reviewed. Bronchoscopy and chest SCT were used to confirm the diagnosis and determine the location of fistula or stenosis. Metallic airway stents were manufactured by Nanjing Micro-Tech Medical Company (Nanjing, China), and were woven with a nickel–titanium alloy wires (Fig. 1). The airway stents were designed according to the diameter and length of the bronchus (Fig. 2, a; Fig. 3, a, b). All the Y shape stent was custom manufactured by Micro-Tech Co. Ltd. (Nanjing, China). [12]

### Stenting procedure

**Fig. 4:**
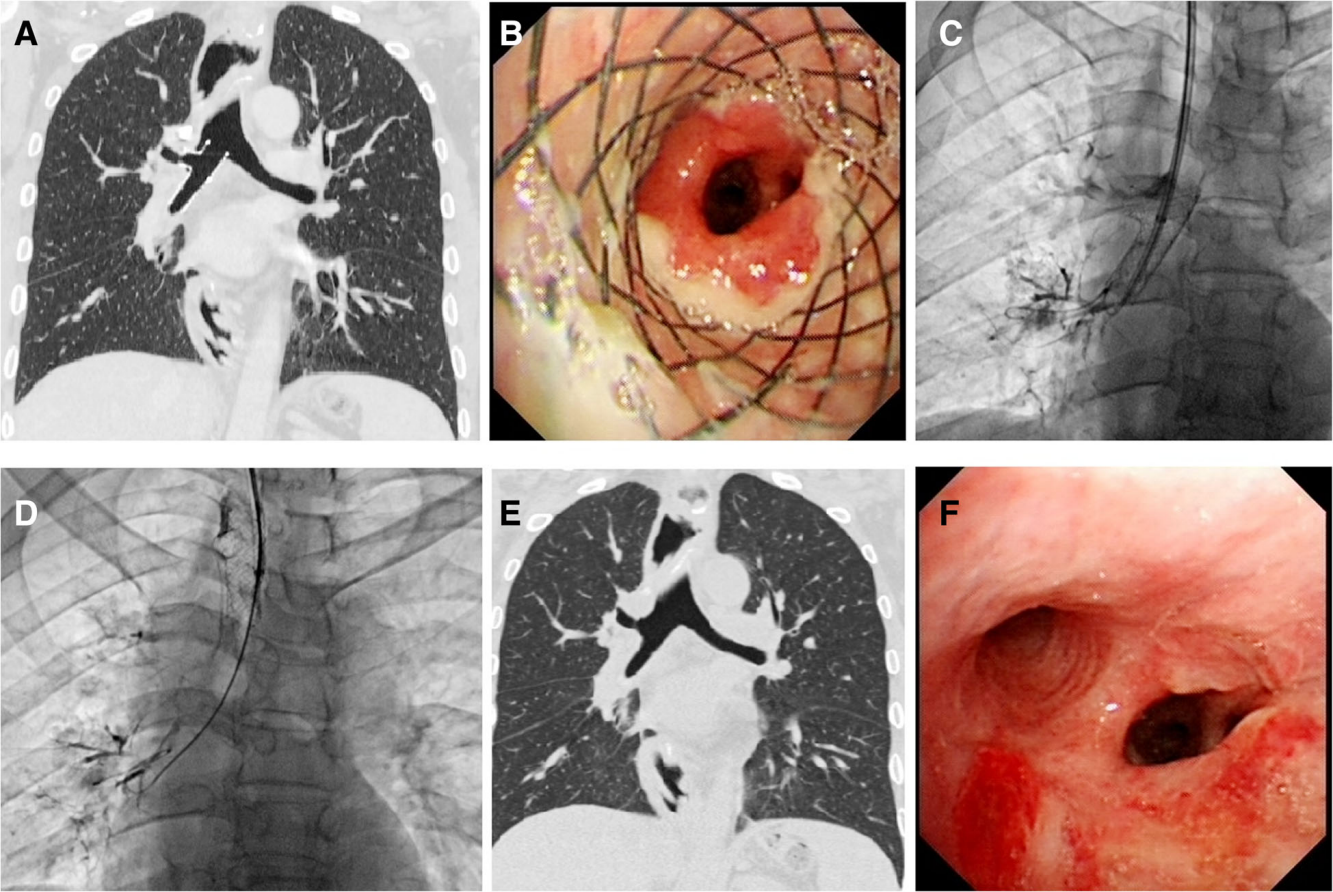
7A 50-year-old man treated by stent placement and removal. a, Airway fistula in right middle lobe bronchus was treated by small y covered stent. b, Bronchoscopy shows sealing of bronchial disease with mild stent restenosis 40 days later. c, Tip of hook placed next to end of stent. d, Metal y stent was successfully withdrawn after 40 days. e, Chest SCT was performed again to confirm sealing of fistula immediately after stent removal. f, Bronchoscopy shows the patency of bronchus after stent removal

The stenting procedure was performed under fluoroscopic guidance [12]. A vertebral artery catheter (Cook Corporation, Bloomington, Ind, USA) was introduced into the distal end of the right lobar bronchi. The location and size of the fistula or stenosis was confirmed by radiography. Then two stiff guide wires (Cook Corporation) were inserted into the distal end of the lobar bronchus. The small y stent was introduced into the primary right carina. The branches of the small y stent were deployed first, and the main body of the stent was deployed at the right main bronchus (Fig. 2, b). For stent removal, a long sheath and retrieval hook was inserted (Fig. 2, e; Fig. 4, c). The tip of the hook was placed next to the end of the stent and withdrawn from the airway wall (Fig. 2, f; Fig. 4, d). Radiography was performed again to show sealing of the fistula or patency of the stent immediately after stenting (Fig. 4, e).

### Follow up

During follow up, bronchoscopy and chest SCT were used to show sealing of the bronchial fistula and stent patency, and radiography was performed if necessary (Fig. 2, c, d; Fig. 3, c, D; Fig. 4, f). Stent removal was conducted once a severe complication was found or patients were cured.

### Statistical analysis

Data were expressed as mean ± standard deviation, and analyzed by ANOVA. Qualitative data were expressed in percentage. Survival rate was compared by Log-rank (Mantel-Cox) Test (GraphPad Software, Inc., USA). Statistical significance was considered when *p* < 0.05.

## Results

### Patient characteristics

Twenty-two patients were diagnosed with esophageal cancer; 4 patients, with lung cancer; and 2 patients, with tuberculosis or pulmonitis. Twenty-two patients underwent lobectomy due to lung cancer (*n* = 1) or resection of esophageal carcinoma (*n* = 21). After a median interval of 1.5 month, patients exhibited airway symptoms, with a median symptom duration of 1.0 month. Thoracogastric airway fistula (71.4%) was the main indication for stent placement in this study. Other indications included airway stenosis and bronchopleural fistula (Table 1).

**Table 1:** The patients’ characteristics

Characteristics	Median (IQR) or No. (%)
Patients, No.	28
Age, years	59 (51–64.5)
Male gender	22 (78.6%)
Duration of symptom, Months	1.0 (0.4–4.8)
Interval between surgery and symptom, Months	1.5 (0.5–13.8)
Duration of stenting procedure, Minutes	31.5 (19.5–44.3)
Previous disease	
Esophageal cancer	22 (78.6%)
Lung cancer	4 (14.3%)
Tuberculosis/pulmonitis	2 (7.1%)
Indications for stent placement	
Airway stenosis	5 (17.9%)
Thoracogastric airway fistula	20 (71.4%)
Bronchopleural fistula	3 (10.7%)
Location of fistula/stenosis	
Right bronchus	22 (78.6%)
Right middle lobe bronchus	6 (21.4%)

### Stenting procedure

Stents were successfully inserted in 25 patients at the first attempt (89.3%). Three patients underwent a successful second attempt, including 2 migrations during withdrawal of the guide wires and one partial obstruction of the left main bronchus due to stent retraction. A total of 31 small y stents were inserted. The median diameter of the stent is 14 mm for main body (Interquartile range, IQR 12, 16), 10 mm for the limb in the right upper bronchus (IQR 10, 12) and 10 mm for another limb in the right middle bronchus (IQR 8, 13). The median length of stent is 15 mm for the main body (IQR 10, 18), 12 mm for the limb in right upper bronchus (IQR 10, 15) and 20 mm for another limb in the right middle bronchus (IQR 15, 25). Five patients underwent successful stent removal due to stent migration (*n* = 1), restenosis (n = 1) or cure efficacy (*n* = 3), with a median time of 76 days (IQR 25.5, 497.5). All of these stents were removed successfully at the first attempt with no complications.

### Complications

No perioperative deaths or severe complications (massive hemorrhage or airway rupture) occurred during stent placement or removal. A total of 12 major complications occurred in 10 patients (35.7%). Four patients showed stent restenosis after a median time of 65 days. Six patients showed sputum retention after a median time of 10.5 days, and endoscopic sputum aspiration was performed for these patients. Migration of two stents was found during withdrawal of the delivery system. One patient showed stent migration 11 days later due to frequent cough and a second small y stent was inserted after removal of the migrated one. All patients tolerated the stent well and had good palliation of airway symptoms (Table 2).

**Table 2:** Clinic effect and complications of stenting

	N (%)	Days after stenting
Clinic efficacy		
Cure or improved	8 (28.6%)	31.8 (25.6, 34.4)
Death	18 (64.3%)	9 (3.9, 53.4)
Loss of follow	2 (7.1%)	–
Complications		
Stent restenosis	4 (14.3%)	65 (43.5, 186.3)
Bronchus obstruction	1 (3.6%)	0
Stent migration	3 (10.7%)	0 (0,11)
Retention of sputum	6 (21.4%)	1.5 (5.3, 169.3)

### Follow up

Two patients were lost to follow up. The remaining 26 patients were followed up for a median time of 9.2 months (IQR 3.5–23.8 months). Seventeen patients died of tumors and one died of myocardial infarction during follow up. The 1-, 3-, and 5-year survival rates were 49.3, 19.6 and 19.6%, respectively. The median survival was 12 months.

## Discussion

Airway stents of various types and materials have been used for the treatment of airway disease, such as silicone stents [15–17] and metallic stents [1–9, 11, 12]. Small y stent placement may be applicable for patients with bronchial disease around the primary right carina. For example, the silicone y stent has been used for the treatment of airway disease around the left carina [13]. However, few studies have reported the use of metallic y stents for the treatment of bronchial disease around the primary right carina. Our study demonstrated that this technique is safe and feasible for these patients with good efficacy. In our study, stents were successfully inserted in all patients with no perioperative death or severe complications, such as massive hemorrhage or airway rupture. Twelve complications occurred in 10 patients. Stent restenosis and sputum retention were the most common complications, occurring in 4 and 6 patients, respectively. All of these complications were successfully treated by endoscopy or re-intervention.

Owing to easy removal, durability and low cost, the silicone stent is widely used clinically [13, 18–20]. However, metallic stents show good support and flexibility, can be placed with the help of guide wires, may be less traumatizing and minimize the procedures in cases with a fistula [14]. Currently, individualized metallic stents are available and can be produce upon request [1, 7, 21]. Metallic large Y stents were designed to be inserted for airway disease at the main carina. Owing to a low rate of stent migration, the metallic large Y stent placement on the primary carina may be an alternative to conventional straight stent placement. In this study, metallic small y stents were customized and used for airway disease around the primary right carina. According to our experience, metallic small y stent placement can be an alternative to silicone y stenting for airway disease around the primary right carina. In addition, implanted Y stents show excellent stability, with a low migration rate [22].

Oki et al. reported 3 cases of malignant disease treated with silicone y stent placement around the primary right carina [17]. The sample size of our study is significantly larger than that of his study, which helps to provide more reliable clinical experience. Metallic stent placement under fluoroscopic guidance dose not require the complex skills needed for rigid bronchoscopy, and this will contribute to the popularization and application of this technology. It needs to be pointed out that silicone stents have to be placed under rigid bronchoscopy only. In addition, rigid bronchoscopy is not only a way to access the airway but also a tool to achieve tumor debulking and dilation of stenosis.

The limitation of this study is its small retrospective nature. A larger prospective study is necessary to further investigate outcomes. In addition, good results of this study might not be reproduced at a less treatment experienced center. In conclusion, metallic small y stent placement is technically feasible, effective and safe for bronchial disease around primary right carina.

## Conclusions

Metallic small y stent placement is technically feasible, effective and safe for bronchial disease around the primary right carina.

## Abbreviations

IQR: interquartile range
SCT: spiral computed tomography
